# Coordinated attenuation of oxidative stress metabolism and alterations of root exudates in maize (*Zea mays* L.) induced by *Streptomyces* rhizobacteria

**DOI:** 10.1007/s10482-026-02372-0

**Published:** 2026-07-08

**Authors:** Juan Fernández, Julia Budelon Gonçalves, Mariana Vieira Franções, Leandro Vieira Astarita

**Affiliations:** Plant Biotechnology Laboratory, School of Health and Life Sciences, Pontifícia Universidade Católica Do Rio Grande Do Sul - PUCRS, Av. Ipiranga, 6681, Partenon, Porto Alegre, RS 90619-900 Brazil

**Keywords:** PGPR, Plant exudates, Enzymatic metabolism, Phenolic compounds, Actinomycetota

## Abstract

Interactions between plants and plant growth-promoting rhizobacteria (PGPR) are critically important for the healthy development of plants. Several *Streptomyces* species stand out as PGPR and biological control agents. This study evaluated the effects of the interaction between maize plants and four *Streptomyces* strains on oxidative stress metabolism and root exudate profiles. Plants treated with strains CLV16, CLV95, CLV104, and CLV179 showed a marked reduction in antioxidant enzyme activities. In shoots, ascorbate peroxidase activity decreased by up to 99% at 15 days compared to control, while catalase and guaiacol peroxidase activities in roots were reduced by approximately 70% and 40–65%, respectively. Root exudate profiling revealed a shift from defense-related specialized metabolites toward primary metabolites. Total amino acids increased up to 2.3-fold (reaching 187 µg/mg root dry mass in CLV104-treated plants), whereas total sugars and phenolic compounds decreased by up to 60% and 70%, respectively. Five-day-old plants exuded the highest levels of caffeic acid when treated with *Streptomyces* isolates, while 15-day-old plants showed higher levels of hydroxybenzoic acid, vanillic acid, and the flavonoid rutin in CLV16 and CLV179 treatments. Notably, the CLV104 strain promoted the lowest exudation of phenolic compounds with potential antimicrobial activity, including caffeic acid (reduced by approximately 92% at 15 days) and undetectable levels of coumarin and cinnamic acid. These findings indicate that *Streptomyces* may cause a coordinated suppression of oxidative defense metabolism alongside alterations of root exudation patterns, with strain-specific effects that may favor beneficial rhizosphere interactions.

## Introduction

Understanding the complex relationships between plants and beneficial rhizobacteria represents a challenge for the development of bioproducts with applications in agriculture. Bioproducts using live microorganisms can promote growth and give defensive advantages to plants. It may also act by increasing the availability of nutrients in the soil, such as phosphates and iron (Medeiros and Lopes 2006). In general, microbial-based bioproducts are formulated using isolates with more than one growth-promoting characteristic (Bashan et al. 2014), such as the synthesis of plant growth regulators (PGR), like enzymes and phytohormones, that improve plant development, as well as nitrogen fixation and phosphate solubilization.

In this context, the use of the rhizobacterium *Azospirillum brasiliense* in bioproducts targeting maize is well established, with numerous commercial formulations already available. (Barbosa and Rocha 2022; Oliveira et al. 2018). *A. brasiliense* promotes nitrogen availability and mitigates water stress in maize, in addition to producing PGR such as auxins, gibberellins, and cytokinins. This capacity to influence plant physiology is a key feature of PGPR (Marques et al. 2020). Indeed, bacterial-based products can actively modulate hormone synthesis in plants (Park et al. 2017). For instance, inoculation of *Bacillus aryabhattai* in soybean plants promotes the synthesis of jasmonic and abscisic acids (Park et al. 2017). Likewise, the Auras® product, containing *B. aryabhattai*, has been successfully used to avoid agricultural losses caused by water stress (Bittencourt et al. 2023).

Among the various bacterial groups with beneficial effects on plants, the phylum Actinomycetota stands out due to the presence of numerous species with PGPR traits. Within this group, *Streptomyces* is the genus with the greatest capacity to produce biologically active metabolites (Alam et al. 2022; Beaulieu 2001). *Streptomyces* are Gram-positive, aerobic bacteria characterised by a complex, filamentous life cycle. Their cell structure involves the formation of chains of cells organized in vegetative hyphae (which colonize the substrate) and aerial hyphae (which differentiate into spores) (Sousa and Olivares 2016). This intricate developmental pattern, transitioning between vegetative growth and spore formation, requires a highly regulated and metabolically demanding process. Consequently, this complex life cycle contributes to the synthesis of a vast and highly diverse array of specialized metabolites (Olanrewaju and Babalola 2018). The compounds found in *Streptomyces* are famously used as antibiotics, insecticides, and herbicides (Kim et al. 2020; Tanaka and Omura 1993). The agricultural potential of this genus extends to the promotion of plant growth, as the example of *S*. *alfalfae* that produces auxins and iron chelators, promoting germination and plant growth (Pang et al. 2022). The synthesis of metabolites of interest in *Streptomyces*, such as auxins and phosphate solubilizers, can be increased under salt stress conditions (Sadeghi et al. 2012), acting as a PGPR and increasing salt stress tolerance in maize (Nozari et al. 2021). This beneficial trait, during saline stress conditions, is also observed in *S*. *venezuelae* for wheat and tomato, indicating the versatility of the genus (Gong et al. 2020).

Biological products with *Streptomyces* are mainly composed of antimicrobial metabolites used to control phytopathogens (Vurukonda et al. 2018). Commercial examples include live *Streptomyces* strains capable of colonizing plants, such as Mycostop® (*S. griseoviridis*) and Actinovate® (*S. lydicus*), which are effective in suppressing phytopathogenic fungi, including *Pythium* spp., *Fusarium* spp., *Rhizoctonia* spp., and *Phytophthora* spp. (Elliott et al 2009; Lahdenperä et al 1991; Minuto et al 2006). These *Streptomyces* initiate an infectious process in the roots, competing with phytopathogens and taking their place. Considering the wide diversity of compounds produced by *Streptomyces*, bacterial strains capable of synthesizing antibiotics and plant growth promoting molecules can be selected, highlighting the potential of this genus to compose formulations of biological products (Reddy et al. 2016).

Although several studies have reported the PGPR potential of live *Streptomyces* strains, the specific biochemical mechanisms by which these bacteria modulate plant root exudation and oxidative stress metabolism remain incompletely characterized (Van der Meij et al. 2017; Mattei et al. 2026). Recent evidence indicates that *Streptomyces* colonization can differentially reprogram root exudate profiles in a strain-dependent manner, affecting the balance between primary metabolites such as amino acids and defense-related specialized metabolites including phenolics and flavonoids (Carlucci et al. 2023). Furthermore, omics-based approaches have revealed that *Streptomyces* spp. modulate plant redox metabolism by altering the activity of antioxidant enzymes such as APX, CAT, and POX, thereby influencing the plant’s capacity to manage reactive oxygen species during early colonization (Mattei et al. 2025). Understanding these biochemical dialogues is essential, as the plant growth-promoting effects of rhizobacteria depend not only on the metabolic profile of the microorganism but also on the plant’s species-specific metabolic responses (Präg et al. 2014; Salla et al. 2016).

Thus, different *Streptomyces*, such as *S*. *coelicolor*, *S*. *griseus*, *S*. *albus*, *S*. *antibioticus* and *S*. *champavatii*, have shown the ability to modulate the enzymatic responses related to oxidative stress in tomato plants (*Solanum lycopersicum* L.) during *Rhizoctonia solani* pathogeny (Singh et al. 2016). In addition to modulating oxidative stress enzyme activity, different *Streptomyces* also can elicit a preventive plant response (Baz et al. 2012). Plants previously treated with *Streptomyces*, capable of promoting the increase of Ca^2+^ in the cytoplasm, modulate the defense signaling and the oxidative explosion of the plant, leading to resistance against the pathogen *Pectobacterium* (Baz et al. 2012). This modulation of enzyme activity can enhance peroxidase and catalase activities, which act on the detoxification of ROS (Zia et al. 2019). In response to the potential benefits of associations between beneficial bacteria and roots, plants can influence the microbial community of the rhizosphere through the exudation of chemical compounds (Cardoso and Andreote 2016). The interaction between plants and microorganisms is species-dependent and can be beneficial for plant development. This interaction starts with the secretion of exudates by the plant roots, promoting an increase in the population of certain microorganisms in the rhizosphere (Berg and Smalla 2009) and the consequent decrease in microbial diversity. However, this decrease in diversity can be offset by greater metabolic activity when compared to other microbial communities farther from the rhizosphere (Zhou et al. 2020).

Rhizosphere microbial diversity has a part in processes that aid in plant development, such as the catalysis of organic compounds, nitrogen fixation, ion solubilization and protection against pathogens and pests (Chaparro et al. 2013). Both the composition of the rhizosphere microbiota and the activities performed by microorganisms can be altered by plant stress responses (Cardoso and Andreote 2016). Plant-microorganism interaction occurs through organic compounds present in plant exudates. In the case of roots, the secreted compounds are quite varied, including large-mass molecules, such as polysaccharides and proteins, and smaller-mass molecules, such as carbohydrates, amino acids, and organic acids (Iannucci et al. 2021).

It is estimated that up to 40% of the total organic carbon synthesized by the plant is released into the rhizosphere as rhizodeposit (Jones and Darra 1994). However, the chemical composition of these root exudates is not static; rather, it is dynamically altered in response to both biotic and abiotic stresses. In an example of biotic stress, cotton plants under the pathogeny of the fungus *Verticillium dahliae* altered the composition of their rhizosphere microbial community due to changes in the synthesis of root exudates (Trivedi et al. 2012).

Similarly, abiotic stress from phosphorus deficiency in the soil promotes an increased exudation of strigolactone in *Medicago truncatulata*, *Sorghum*, and *Oryza sativa* plants (Nasir et al. 2019; Schlemper et al. 2017). This specific exudation is considered an adaptive mechanism, as the metabolite promotes the colonization of the rhizosphere by mycorrhizae, which subsequently mitigate the nutrient deficiency (Banasiak et al. 2020).

Root exudate diversity depends on the growth phase of the plant and its interactions in the rhizosphere. Maize roots can reduce carbohydrate exudation when under *Agrobacterium* pathogeny (Laheurte et al. 1990). However, roots under the influence of the fungus *Laccaria laccata* exude more total carbohydrates and amino acids (Hale and Moore 1979; Laheurte et al. 1990). These alterations in the exudate profile may be caused by changes in cellular permeability due to phytopathogens, leading to greater loss of carbohydrates. On the other hand, bacterial phenazines can stimulate the exudation of amino acids by the roots, favoring microbial growth in the rhizosphere (Phillips et al. 2006). Likewise, bacterial auxins, such as indole-3-acetic acid (IAA), stimulate the exudation of sugars by the roots (Wittenmayer and Merbach 2005). On the other hand, phenolic acids present in the exudates can help in the availability of soil nutrients and in the acclimatization of plants to stresses (Juszczuk et al. 2004). Roots can attract different beneficial microorganisms through the exudation of compounds, such as benzoxazinoids produced by grasses, capable of attracting the rhizobacterium *Pseudomonas putida* (Neal et al. 2012). In this sense, there is a preference of rhizobacteria for aromatic organic acids, such as nicotinic, shikimic, salicylic, cinnamic and IAA (Zhalnina et al. 2018). Arabinogalactan proteins exuded by the roots, mainly in the root cap region, can act as signals in the interaction of the plant with different PGPR (Nguema-Ona et al. 2013). The metabolites secreted by roots can be passively or actively released into the rhizosphere. Carbohydrates are released by diffusion, while strigolactone and phenolic compounds are actively exuded by membrane transporters (Badri et al. 2012; Maurer et al. 2021).

Therefore, root exudates play an important role in the success of the plant-microorganism interactions, enabling the establishment in the rhizosphere of bacteria that help modulate both responses to oxidative stress and plant growth. Although *Streptomyces* are recognized for their ability to produce bioactive compounds and promote plant growth, the specific effects of live *Streptomyces* colonization on the modulation of root exudate profiles, particularly regarding the balance between primary metabolites (sugars, amino acids) and defense-related specialized metabolites (phenolics, flavonoids), remain poorly understood (Van der Meij et al. 2017). Furthermore, it is unclear whether changes in root exudation are coordinated with alterations in the plant’s oxidative stress metabolism during early stages of interaction. To address this gap, we hypothesized that colonization by *Streptomyces* would attenuate the activity of oxidative stress enzymes (APX, CAT, POX) in maize tissues and simultaneously reprogram root exudate profiles toward increased release of primary metabolites (amino acids) and reduced exudation of defense-related specialized metabolites (phenolics, flavonoids), with strain-specific outcomes depending on the PGPR traits of each isolate. To test this hypothesis, this work aimed to evaluate the metabolic responses of young maize plants upon interaction with four *Streptomyces* strains, focusing on antioxidant enzyme activities and the compositional dynamics of root exudates.

## Materials and methods

*Streptomyces* rhizobacteria was obtained from the collection of microorganisms at the Laboratory of Plant Biotechnology at PUCRS (Pontifical Catholic University of Rio Grande do Sul, Brazil). Briefly, the bacteria from the collection were isolated from rhizospheric soil through cultivation in selective ISP2 medium (Dalmas et al 2011; Shirling and Gottlieb 1966). The rhizobacteria used were isolated from root samples of different plants: CLV16—*Cortaderia selloana* (Poaceae) (S 29° 29.664 W 050°11.512), CLV95—*Brachiaria* sp. (Poaceae) (S 22°28′19,33’’ W 54°48′30,83’’), CLV104—*Phaseolus vulgaris* (Fabaceae) (S 14°59′9,43’’ W 54°4′35,78’’) and CLV179—*Cucumis melo* (Cucurbitaceae) (S 4° 46′ 30″ W 37°14′45"). The selected isolates were stored at -80 ºC (ISP2 medium: glycerol; 1:1 v/v). Subsequently, the rhizobacterial isolates were recovered in liquid culture medium ISP2 or ISP4 for 5 days and under controlled temperature and agitation (26 °C, 120 rpm) (International *Streptomyces* Project) (Hong et al. 2007) and used in the experiments. The 16S rDNA sequencing confirmed the genus *Streptomyces*, but it was not possible to identify it at the species level. The sequences are deposited in GenBank CLV16 (ON723945.1), CLV95 (MN461005.1), CLV104 (ON723950.1), and CLV179 (MN461009.1). Seeds of maize (*Zea mays* L.), cultivar BRS 1015, were used in this study. The seeds were obtained from an authorized commercial source in Brazil, corresponding to a cultivar developed and distributed by Embrapa (Brazilian Agricultural Research Corporation). The plant material was not collected from natural or wild populations and therefore no geographic coordinates are applicable. All plants were cultivated under controlled experimental conditions for the purposes of this study.

### Culture media, reagents, and cultivation of Streptomyces

*Streptomyces* isolates were cultivated in triplicates. CLV16 cultivation was carried out using ISP2 medium, while the isolates CLV95, CLV104 and CLV179 were cultivated in ISP4. Both culture media are referenced in International *Streptomyces* Project (Shirling and Gottlieb 1966). ISP2 medium consisted of 4 g L^−1^ yeast extract, 4 g L^−1^ glucose, and 10 g L^−1^ malt extract, pH 7. ISP4 medium contained soluble starch (10 g L^−1^), K_2_HPO_4_ (1 g L^−1^), MgSO_4_·7H_2_O (1 g L^−1^), NaCl (1 g L^−1^), (NH_4_)_2_SO_4_ (2 g L^−1^), CaCO₃ (2 g L^−1^), FeSO_4_·7H_2_O (1 mg L^−1^), MnCl_2_ (1 mg L^−1^), and ZnSO_4_·7H_2_O (1 mg L^−1^), with pH between 7.0 and 7.3.

### Cultivation and inoculation of rhizobacteria in plants

The isolates used in this study were previously selected based on their PGPR traits, including indolic compound production (CLV95 and CLV104), nutrient mobilization (CLV16 and CLV95), and plant growth promotion under greenhouse conditions (CLV104). In contrast, CLV179 was intentionally included as a non-PGPR reference strain, as it did not exhibit detectable indolic production, nutrient mobilization traits, or plant growth–promoting effects in previous assays. The strains CLV16, CLV95 and CLV104 were cultivated in ISP2 liquid medium and CLV179 in ISP4 liquid medium (Kuster 1972; Shirling and Gottlieb 1966). The rhizobacteria were cultivated for 5 days at 26 °C, as this temperature is considered optimal for the growth and metabolism of these specific isolates, under constant agitation (140 rpm). Subsequently, the dense mycelial structures formed during cultivation were disaggregated (Zacchetti et al. 2018) and the bacterial suspension homogenized through filtration on a plastic screen (1 mm mesh) to remove large particles and aggregates, ensuring a uniform bacterial suspension. After this step, the bacterial suspension was serially diluted, for the determination of colony forming units (CFU/mL), in solid culture medium ISP2 or ISP4.

The homogenized bacterial suspension was used for the inoculation of maize seeds. Treatments were divided using 40 seeds each and soaked in contact with the bacterial suspensions for 24 h. Inoculum concentrations were not standardized across the treatments. Instead, each strain was applied at its maximum growth under the selected culture conditions. This approach was adopted to better reflect practical on-farm applications, where microbial inoculants are typically used based on culture performance rather than normalized CFU counts. Thus, treatments were applied in the following concentrations: CLV16 (1.9 × 10^5^ CFU/mL), CLV95 (2.6 × 10^7^ CFU/mL), CLV104 (7.6 × 10^7^ CFU/mL) and CLV179 (1,1 × 10^6^ CFU/mL). Control treatment consisted of seed soaked in sterile water for the same amount of time. All treatments were maintained under agitation (140 rpm). To increase the adhesion of bacteria to the surface of the seeds, all treatments were supplemented with 1% (v/v) of the surfactant Silwet® L-77.

After the inoculation phase, the seeds were sown in pots containing sterile vermiculite substrate and maintained in a plant-growth room under controlled conditions (25 °C, 16 h photoperiod) for 5 or 15 days. The 5-day time point represents the early post-emergence stage, when initial root colonization by *Streptomyces* is actively occurring, while the 15-day time point corresponds to a more established vegetative stage, allowing assessment of sustained physiological effects. This time points were selected based on preliminary experiments indicating significant changes in antioxidant enzyme activities and exudate profiles within this developmental window. Plants were grown without nutrient supplementation, receiving water only on an every-other-day basis. The experiment was conducted as a completely randomized design with four biological replicates per treatment, each replicate consisting of a pool of 15 plants (for enzymatic and exudate analyses) or 5 plants (for dry mass determination). The entire experiment was independently repeated twice to ensure reproducibility, yielding consistent results across replicates.

### Enzymatic activity

Leaves and roots were sampled from maize plants at 5 and 15 days post-germination, using 15 plants per treatment for each time point. Subsamples of 0.5 g were obtained from a pool of plant matter from different plants within the treatment and submitted to different analyses. Total soluble proteins were analysed using the Bradford method (Bradford 1976). Tissues were extracted using 50 mM sodium phosphate buffer (pH 7), 0.05 g/L Polyvinylpyrrolidone (PVP) and 30 µL/L Triton X-100. The determinations were made in microplates with the aid of the Spectramax 190 Multimode Microplate Reader (Molecular Devices) at an absorbance of 595 nm. The calibration curve for proteins was performed using bovine serum albumin, which resulted in y = 0.3008x + 0.0247, with ‘x’ being the protein concentration in mg/mL and ‘y’ being the absorbance. The ‘r’ value was 0.9197.

Ascorbate peroxidase (APX; EC 1.11.1.11) activity was determined after extraction of tissues using 500 mM potassium phosphate buffer (pH 7.0), according to Amako et al. (1994). The colorimetric reaction was read in a spectrophotometer (UV-1600 Spectrophotometer) at 290 nm using ascorbic acid and H_2_O_2_ in the reaction. Enzymatic activity was evaluated by hydrogen peroxide decay over 60 s. The following equation was used to calculate the enzymatic activity of APX: −[slope/((2800 µM) (µL sample) (mg protein/mL))] × 10^12^. Slope calculated as time by Log10. The constant 2800 refers to the molar extinction coefficient of ascorbate (ε_290 nm_ = 2,8 mM^−1^ cm^−1^).

Catalase enzyme activity (CAT; EC 1.11.1.6) was determined in 500 mM Potassium Phosphate buffer (pH 7.0) (Aebi 1974). The reaction was read in a spectrophotometer (UV-1600 Spectrophotometer) at 240 nm over 56 s. The following equation was used to calculate the enzymatic activity of CAT: −[slope/(4.6 × 10^7^) (µL sample in the cuvette) (mg protein/mL))] × 10^12^. Slope calculated as time by Log10. The constant 4.6 × 10^7^ refers to the H_2_O_2_ molar extinction coefficient (ε_240 nm_ = 46 M^−1^ cm^−1^).

The determination of polyphenoloxidase activity (POX-Guaiacol; EC 1.11.1.7) was performed by extracting tissues with 50 mM sodium phosphate buffer (pH 6) (Amako et al. 1994; Tekchandani and Guruprasad 1998). The reaction was read in a spectrophotometer (UV-1600 Spectrophotometer) at 420 nm using H_2_O_2_ and Guaiacol for readings over 30 s. For the calculation, the following equation was used: [sample absorbance/(26,600 µM) (µL sample) (mg protein/mL))] × 10^6^/25. The constant 26,600 refers to the molar extinction coefficient of guaiacol (ε_420 nm_ = 26,6 mM^−1 ^cm^−1^). Specific enzymatic activity was expressed as nKatal/mg of protein. One katal (kat) corresponds to the amount of enzymatic activity that transforms 1 mol s^−1^ of substrate. All measurements were performed at controlled temperature (25 °C).

### Maize plants root exudates

Root exudates were collected using a short-term aqueous system to minimize background interference and enable accurate detection of released metabolites under controlled conditions. Bacterial suspensions CLV16, CLV95, CLV104 and CLV179 were inoculated into maize seeds, following the methodology previously described. After the inoculation phase, the seeds were transferred and kept for 5 and 15 days in a sterile vermiculite substrate, under controlled temperature (25 °C) and photo period (16 h).

After the growth period, plants were carefully removed from the vermiculite substrate, and roots were gently washed with sterile distilled water to remove adhering particles. Plant tissues (0.5 g) were then transferred to sterile flasks containing filtered sterilized water (0.22 µm membrane filter), with roots fully submerged. Volumes were adjusted according to plant age: 30 mL for 5-day-old plants and 100 mL for 15-day-old plants. To prevent microbial contamination during exudate collection, flasks were wrapped in aluminum foil to exclude light and maintained under the same controlled conditions (25 °C, 16 h photoperiod) for 24 h. After collecting, the root bathing solution was filtered under vacuum through Whatman No. 1440 filter paper to remove root debris and particulate matter. Roots were then excised, dried at 60 °C until constant weight, and weighed for biomass determination. The filtrate was lyophilized (Terroni, LS 3000), and the resulting exudate concentrate was stored at –20 °C until analysis. Prior to quantification, lyophilized material was resuspended in 3 mL of ultrapure water (Merck, USA). All exudate data were normalized by root dry mass (expressed as µg per mg root dry mass) to account for variations in root biomass among treatments and developmental stages.

Samples were analysed by colorimetry for reducing sugars, amino acids, phenolic compounds, and flavonoids. Total reducing sugars were analysed by the Antrona method (Trevelyan et al. 1952) and the reactions read on microplates using Spectramax at 620 nm. Total amino acids were analysed by the Ninhydrin method (Yemm and Ocking 1955) and reactions were read at 570 nm. Total phenolic compounds were analysed using the Folin–Ciocâlteu reagent (Singleton et al. 1999). Reactions were read at 765 nm. Total flavonoids were analysed using aluminum nitrate (Mammen and Daniel 2012) and reactions read at 415 nm.

Phenolic and flavonoid compounds profile was analysed using a high-performance liquid chromatograph (HPLC) equipped with a MetaSil ODS C18 column (5 µm; 250 × 4.6 mm), with a C18 pre-column and Clarity Chromatography software. For this purpose, the resuspended samples were filtered through a membrane (0.45 µm) and the phenolic compounds determined using methanol and ultrapure water acidified with 2.5% formic acid as mobile phase, with a flow rate of 0.5 mL/min and temperature of the column set to 25 °C. Quantification was performed using calibration curves of commercial phenolic standards (Sigma-Aldrich®). The gradient used was: 10% [MeOH 100%] (for 10 min), 20% (from 10 to 25 min), 80% (from 25 to 32 min), 100% (from 32 to 37 min) and 10% (at 37 min).

Flavonoid profile determination was carried out using acetonitrile and ultrapure water supplemented with 2% phosphoric acid as mobile phase, with a flow rate of 0.5 mL/min and column temperature adjusted to 40 °C. The quantification of flavonoids was performed using calibration curves established with commercial standards (Sigma-Aldrich®). The gradient used was 98% acetonitrile (100%) for 20 min and 50% (from 20 to 40 min).

### Data analysis

Each treatment (four *Streptomyces* strains plus an uninoculated control) was evaluated in three independent biological experiments conducted at different time points. Within each independent experiment, 15 plants were used per treatment. For enzymatic and root exudate analyses, samples from three individual plants were pooled to generate one analytical replicate. The independent experiments were considered biological replicates for statistical analyses. Experimental repetition was not included as a random factor in the statistical model. All datasets were initially evaluated for normality using the Shapiro–Wilk test. Data meeting parametric assumptions were analyzed using one-way ANOVA followed by Tukey’s post hoc test (*p* ≤ 0.05). Statistical analyses were performed using IBM SPSS Statistics version 20 (USA).

## Results

### Maize plants enzymatic activity

**Fig. 1:**
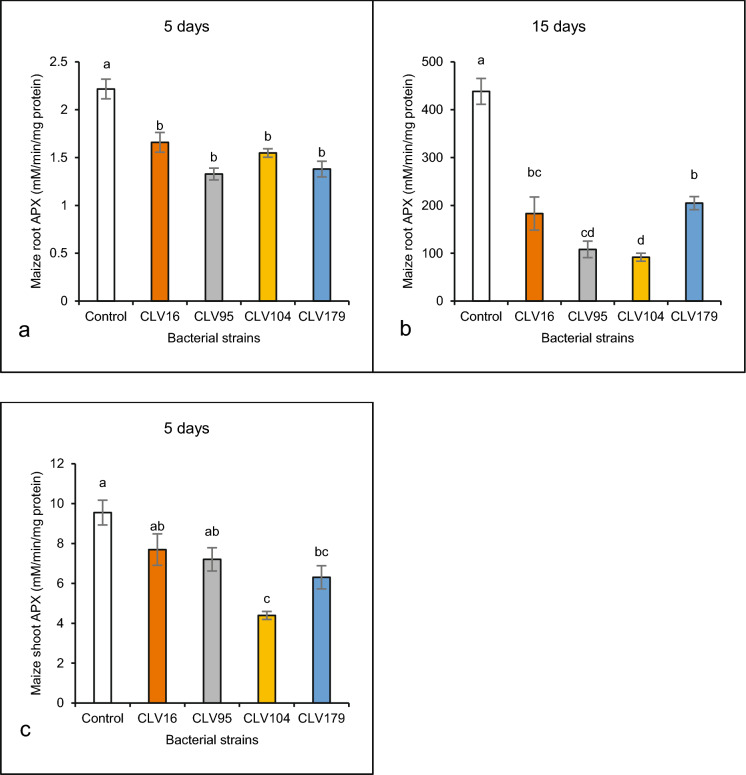
APX enzyme activity in maize plants at 5 and 15 days after germination. Seeds were inoculated with *Streptomyces* strains (CLV16, CLV95, CLV104, and CLV179), with uninoculated seeds used as control.** a** Root tissues at 5 days; **b** root tissues at 15 days; **c** shoot tissues at 5 days. APX activity in shoot tissues at 15 days could not be determined due to technical limitations. Bars represent the standard error of the mean. Different letters indicate statistically significant differences (ANOVA, Tukey’s test, α = 0.05)

APX enzyme activity was higher in shoots of 15-day-old plants than in 5-day-old plants (Fig. 1a, b). It was not possible to determine the root activity of APX in plants with 15 days after germination, due to the lack of repeatability in the assay. The highest activity occurred in the control treatment, both in the 15-day-old (438.27 mM/min/mg) and in the 5-day-old (2.21 mM/min/mg) plants. Regardless of age (5 or 15 days), plants inoculated with *Streptomyces* isolates showed lower APX activity in shoot compared to control (Fig. 1a, b). However, the roots of 5-day-old plants showed a decrease in APX activity only in the treatments with CLV104 (4.39 mM/min/mg) and CLV179 (6.3 mM/min/mg), compared to the control (9.55 mM/min/mg) (Fig. 1c).

**Fig. 2:**
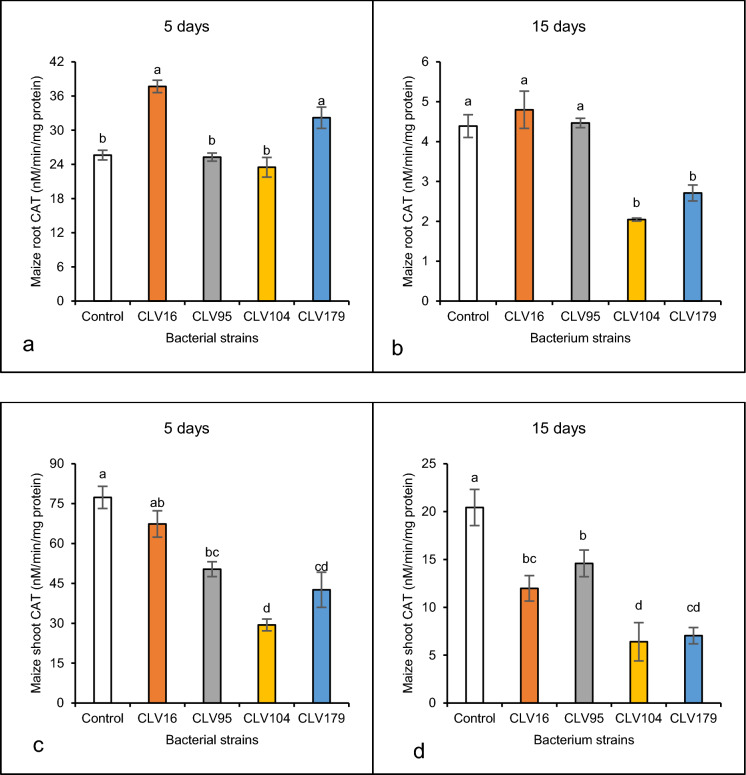
Catalase (CAT) enzyme activity in 5- and 15-day old maize plants. Seeds were inoculated with *Streptomyces* strains (CLV16, CLV95, CLV104, and CLV179), with uninoculated seeds used as control. **a** Root tissues at 5 days; **b** root tissues at 15 days; **c** shoot tissues at 5 days and **d** shoot tissues at 15 days. Bars represent the standard error of the mean. Different letters indicate statistically significant differences (ANOVA, Tukey’s test, α = 0.05)

Highest CAT activity in maize shoots occurred in the CLV16 treatment, with 5 days (37.68 mM/min/mg), with no statistical difference with the CLV179 treatment (32.19 mM/min/mg). While in the roots (Fig. 2c, d), the highest activities occurred in the control with 5 days (77.34 mM/min/mg) and 15 days (20.42 mM/min/mg).

Plants with 15 days of age showed the lowest CAT activities in maize shoot in the treatments with CLV104 (2.0 mM/min/mg) and CLV179 (2.71 mM/min/mg), compared to the control (4.38 mM/min/mg). Likewise, the roots of 15-day-old plants showed a drastic reduction in CAT activity in the CLV104 (6.4 mM/min/mg) and CLV179 (7.03 mM/min/mg) treatments, compared to the control (20 0.42 mM/min/mg) (Fig. 2d). The presence of *Streptomyces* in the roots caused a reduction in CAT activity, regardless of plant age.

**Fig. 3:**
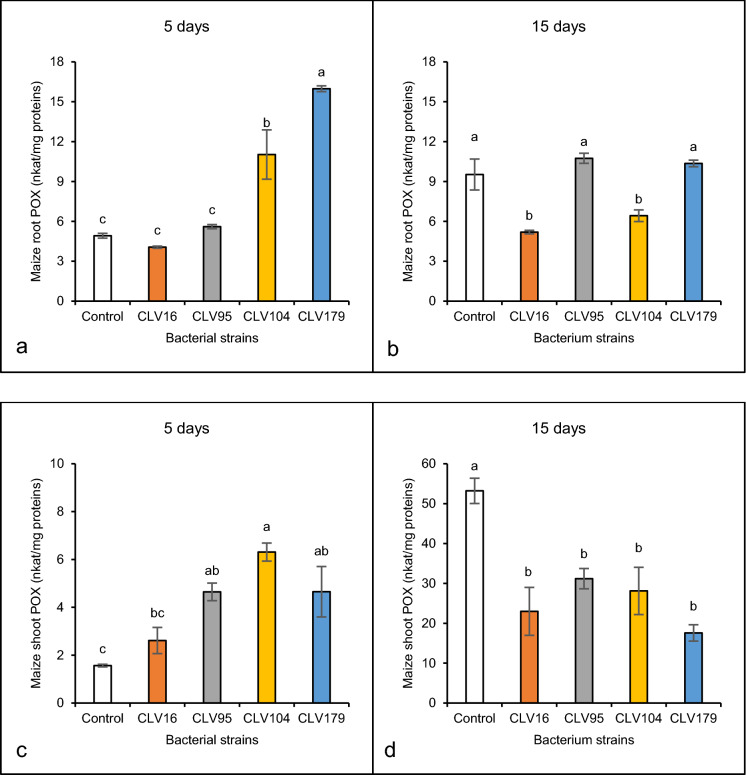
POX enzyme activity in 5- and 15-day old maize plants. Seeds were inoculated with *Streptomyces* strains (CLV16, CLV95, CLV104, and CLV179), with uninoculated seeds used as control. **a** Root tissues at 5 days; **b** root tissues at 15 days; **c** shoot tissues at 5 days and **d** shoot tissues at 15 days. Bars represent the standard error of the mean. Different letters indicate statistically significant differences (ANOVA, Tukey’s test, α = 0.05)

POX activity was higher in maize shoots of 5-day-old plants submitted to CLV104 (11.03 nkat/mg) and CLV179 (15.97 nkat/mg) treatments, compared to control (4.91 nkat/mg) (Fig. 3a). However, in plants with 15 days, there was a decrease in this activity in shoot tissues with CLV104 (6.42 nkat/mg) and CLV16 (5.19 nkat/mg), compared to the control (9.52 nkat/mg) (Fig. 3b).

POX activity in maize roots showed an increase in plants with 5 days, in treatments with CLV 95 (4.64 nkat/mg), CLV104 (6.3 nkat/mg) and CLV179 (4.65 nkat/mg), compared to the control (1.56 nkat/mg) (Fig. 3c). Later, when the plants reached 15 days of age, there was a decrease in POX activity in the roots of plants inoculated with *Streptomyces*. Ranging from 31.18 to 17.57 nkat/mg, compared to control activity (53.21 nkat/mg) (Fig. 3d).

### Root exudates

**Fig. 4:**
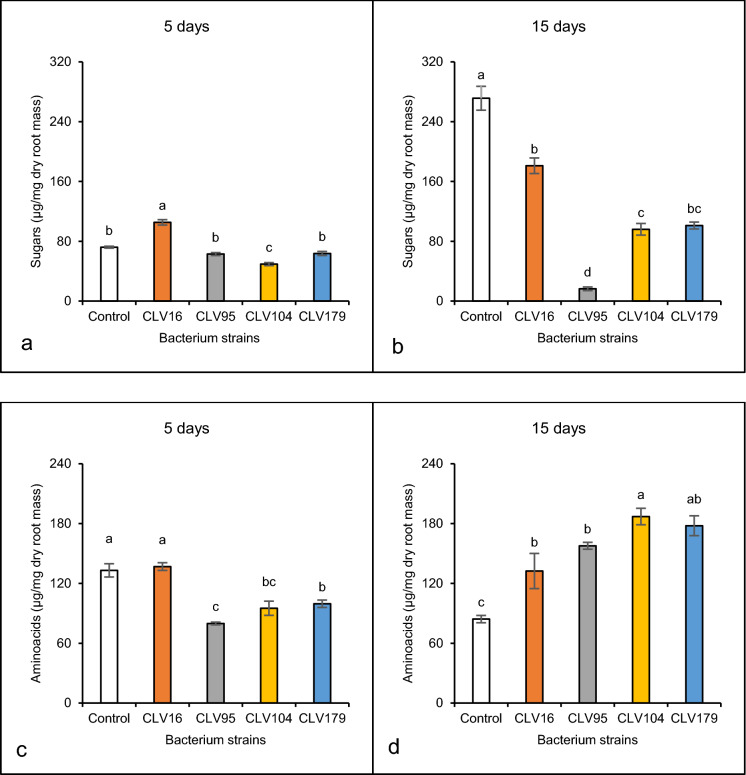
Metabolic profile of root exudates from maize plants. Plants 5- and 15-days-old. Reducing sugars (**a**, **b**) and amino acids (**c**, **d**). Seeds were inoculated with the *Streptomyces* strains (CLV16, CLV95, CLV104 and CLV179). The bars represent the standard error of the mean. Different letters, within the analysed parameter, indicate a statistically significant difference (ANOVA, Tukey, α = 5%)

Evaluations of root exudates indicated that 5-day-old maize plants exuded the highest levels of reducing sugars (105.32 µg/mg) when treated with CLV16 (Fig. 4a). However, plants treated with CLV104 showed the lowest levels of sugar exudates. On the other hand, the presence of *Streptomyces* in the 15-day-old plants promoted a reduction in the concentration of total sugars in the exudates, regardless of the bacterial isolate (Fig. 4b).

Plants treated with CLV95 showed a marked drop in the concentration of sugars in the exudates throughout their development, ranging from 62 µg/mg (at 5 days) and 16 µg/mg (at 15 days) (Fig. 4a).

Total amino acid concentration in root exudates of 5-day-old plants was lower in treatments CLV95 (79.75 µg/mg), CLV104 (95 µg/mg) and CLV179 (99.5 /mg) (Fig. 4c). However, this concentration increased in 15-day-old plants for all treatments (Fig. 4d), reaching the highest concentration in CLV104 (187 µg/mg), with no statistical difference with the CLV179 treatment (177.86 µg/mg).

**Fig. 5:**
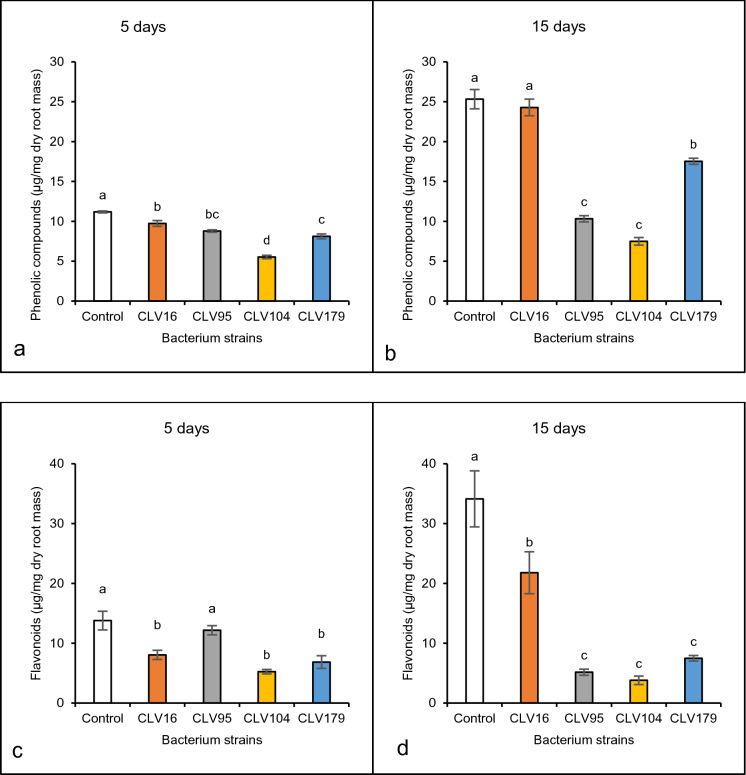
Metabolic profile of root exudates from maize plants. Plants 5- and 15-days-old. Total phenolic compounds (**a**, **b**) and flavonoids (**c**, **d**). Seeds were inoculated with the *Streptomyces* strains (CLV16, CLV95, CLV104 and CLV179). The bars represent the standard error of the mean. Different letters, within the analysed parameter, indicate statistically significant difference (ANOVA, Tukey, α = 5%)

Roots of 5-day-old plants exuded fewer phenolic compounds in the *Streptomyces* treatments (Fig. 5a) compared to the control (11.18 µg/mg). However, 15-day-old plants showed the lowest concentrations of phenolic compounds in the treatments with CLV95 (10.32 µg/mg), CLV104 (7.5 µg/mg) and CLV179 (17.53 µg/mg), compared to the control (25.32 µg/mg) (Fig. 5b).

### Exudate metabolic profile

Plants treated with CLV95 and CLV104 showed the lowest levels of gallic acid, compared to controls 5 or 15 days (Table 1). Hydroxybenzoic acid was only detected in 15-day exudates in control (1.05 ng/mg) and CLV179 (3.22 ng/mg) treatments. Vanillic acid had a higher concentration in the control treatment (174.37 ng/mg) after 5 days and was not detected in the CLV104 treatment. In 15 days, there was a drastic increase in vanillic acid levels, with the highest concentration observed in the exudates of plants treated with CLV179 (542.26 ng/mg) and the lowest levels in CLV104 (43.22 ng/mg). The lowest levels of caffeic acid (262.99 ng/mg) occurred in the CLV104 treatment at 15 days. However, the highest levels of this compound at 15 days were observed in the control (3,238.37 ng/mg) and in CLV16 (2.852,11 ng/mg).

**Table 1:** Specialized metabolite exudates profile from maize plant roots

Metabolites (ng/mg root dry mass)	Control	CLV16	CLV95	CLV104	CLV179
*Plants 5 days*					
Gallic acid	8,63 a	ND	5,98 ab	3,47 b	ND
Hydroxibenzoic acid	ND	ND	ND	ND	ND
Vanillico acid	174,37 a	114,63 ab	57,96 b	ND	74,21 b
Caffeic acid	272,14 c	4811,24 a	2826,99 b	3305,16 ab	3334,21 ab
Ferulic acid	4,22 ab	12,01 a	9,75 ab	ND	1,26 b
Coumaric acid	21,52 ab	ND	33,90 a	16,63 b	13,18 b
Coumarin	ND	ND	ND	ND	ND
Cinnamic acid	ND	ND	ND	ND	ND
Rutin	ND	4280 a	1330 a	1630 a	1980 a
Apigenin	ND	ND	ND	ND	ND
Kaempferol	ND	ND	ND	ND	ND
Quercetin	ND	ND	ND	ND	ND
*Plants 15 days*					
Gallic acid	12,27 a	6,15 ab	4,08 b	3,98 b	4,98 ab
Hydroxibenzoic acid	1,05 b	ND	ND	ND	3,22 a
Vanillic acid	253,61 bc	285,12 b	247,10 bc	43,22 c	542,26 a
Caffeic acid	3238,37 a	2852,11 a	ND	262,99 b	992,39 b
Ferulic acid	3,7 b	12,19 a	3,96 b	3,04 b	2,57 b
Coumaric acid	ND	12,19 b	8,24 bc	1,89 c	22,40 a
Coumarin	13,3 a	ND	9,82 a	ND	9,58 a
Cinnamic acid	7,76 a	5,59 a	2,10 a	ND	1,00 a
Rutin	3200 a	2130 b	220 c	120 c	210 c
Apigenin	ND	ND	ND	ND	ND
Kaempferol	ND	ND	ND	ND	ND
Quercetin	ND	ND	ND	ND	ND

Plants 5- and 15-days-old. Seeds were inoculated with the *Streptomyces* strains (CLV16, 95, 104 and 179). Control, uninoculated seeds. ND was below the detection limit. The total concentration of phenolic compounds was calculated excluding Rutin. Different letters in the row, within the plant age parameter, indicate a statistically significant difference (ANOVA, Tukey, α = 5%)

Notably, 5-day-old plants exuded the highest levels of caffeic acid when treated with *Streptomyces* (ranging from 4811.24 to 2826.99 ng/mg) compared to the control (272.14 ng/mg). On the other hand, at 15 days, the plants with CLV104 showed the lowest levels of this compound (262.99 ng/mg), compared to the control treatment (3,238.37 ng/mg). Ferulic acid levels showed little variation among exudates from plants treated with *Streptomyces*. The concentrations of coumaric acid in the plants treated with the rhizobacterium, in 5 days, were like the control (21.52 ng/mg). However, in 15 days, the lowest levels occurred in the CLV104 (1.89 ng/mg) and CLV95 (8.24 ng/mg) treatment.

Coumarin was not detected in the exudates of 5-day-old plants, being only detected in 15 days in the control (13.3 ng/mg), CLV95 (9.82 ng/mg) and CLV179 (9.58 ng/mg) treatments. Similarly, cinnamic acid (precursor of coumarins) was not detected within 5 days. No coumarin or cinnamic acid was detected in the exudates of plants inoculated with CLV104.

## Discussion

Concentrations of ROS, such as superoxide and hydrogen peroxide, increase in plant tissues under stress. These compounds are inactivated by enzymes such as APX, CAT and POX, which act in different cell compartments. APX and CAT are the main degraders of H_2_O_2_ (Jaleel et al. 2009). APX is found in vascular plants, occurring in the membrane of thylakoids and organelles such as peroxisomes and glyoxysomes, in addition to the cytosol (Bunkelmann and Trelease 1996; Chen and Asada 1989; Yamaguchi et al. 1995). APX uses ascorbate as a H_2_O_2_ reducer inside chloroplasts, being important for the regulation of peroxides in leaves (Kvaratskhelia et al. 1997) and in other plant tissues (Groden and Beck 1979; Nakano and Asada 1981; Shigeoka et al. 1980). The decrease in APX activity corroborates the behavior observed in enzymes related to oxidative stress in plants inoculated with PGPR (Chandran et al. 2021; Jha and Subramanian 2014). Our results indicate that the colonization of the roots by *Streptomyces* isolates led to a decrease in the activity of the APX enzyme in the shoot tissues of 5- and 15-day old maize plants, with emphasis on the plants treated with CLV104 isolate. The absence of APX activity data in roots of 15-day-old plants represents a limitation of the present study, likely due to low enzyme extractability and/or interference from root-derived compounds. However, this gap does not compromise the overall interpretation of oxidative metabolism, as antioxidant responses were consistently evaluated using multiple enzymatic markers, including POX and CAT, which showed coherent trends across treatments. Moreover, APX activity was successfully assessed in other tissues and/or developmental stages, supporting a consistent interpretation of the antioxidant system. Therefore, conclusions were based on an integrated, multi-parameter analysis rather than on a single enzymatic measurement. (Amako et al. 1994). The results also indicate that 15 days after germination all plants responded to the presence of *Streptomyces* by drastically decreasing the activities of CAT and POX enzymes in the roots. This reduction in oxidative stress-related enzyme activities may indicate that plants recognize *Streptomyces* as beneficial organisms (Dalmas et al. 2011). In general, there is an increase in the production of ROS and an oxidative burst as the initial response of the plant to microorganisms (Kumar et al. 2009). In this sense, the increase in POX activity, both in shoot and root, in the 5-day-old plants (Fig. 3a, c), may indicate an increase in H_2_O_2_ production due to the initial colonization of the plant by *Streptomyces* (Nozari et al. 2022).

Guaiacol peroxidase is the main cell wall enzyme responsible for breaking down hydrogen peroxide (Bazzo et al. 2011). In the early stages of plant-beneficial microorganism interaction, ROS is produced in a controlled manner in the apoplast or directly in the rhizosphere (Nanda et al. 2010). The beneficial microorganisms that resist this initial phase, colonize the rhizosphere, and modulate the plant’s ROS production systems (Berrios and Rentsch 2022). Tomato plants treated with beneficial *Streptomyces* showed higher activities of enzymes related to oxidative stress caused by *Rhizoctonia solani* (Singh et al. 2016). These beneficial microorganisms can be useful as a strategy to increase plant resilience to environmental stresses.

While the reduced activities of APX, CAT, and POX observed in our study suggest a state of diminished oxidative stress following *Streptomyces* colonization, we acknowledge that alternative interpretations are possible. In principle, decreased antioxidant enzyme activity could also reflect suppression of basal plant defense mechanisms, potentially compromising the plant’s ability to respond to subsequent stresses. However, several lines of evidence support the interpretation of a beneficial interaction. First, the attenuation of enzyme activities was accompanied by a coordinated alteration of root exudates, increased amino acid release and reduced exudation of defense-related phenolics, a pattern consistent with the establishment of a mutualistic rhizosphere environment (Sasse et al. 2018; Van der Meij et al. 2017). Second, colonized plants exhibited no visible signs of stress or growth impairment under our experimental conditions. Third, similar reductions in antioxidant enzyme activities have been documented in plants associated with beneficial *Streptomyces* strains and were correlated with enhanced tolerance to subsequent biotic and abiotic stresses (Singh et al. 2016; Nozari et al. 2021). Nevertheless, direct quantification of ROS accumulation and transcriptional analysis of defense-related genes would provide stronger evidence for the nature of this response. Future studies integrating these approaches will be valuable to fully elucidate whether the observed attenuation represents a genuine stress-mitigating response or a suppression of basal defenses.

Root exudation is an adaptive strategy that enables plants to interact with the environment and optimize their growth and survival. Amino acids, like sugars released by roots, can be used by rhizosphere microorganisms (Bacilio-Jiménez et al. 2003). It has been observed in *Pseudomonas* that root colonization by these beneficial rhizobacteria may be dependent on exudation of specific amino acids such as L-tryptophan and L-arginine (Simons et al. 1997). Increasing the concentration of amino acids in the rhizosphere reduces dissolved organic carbon and increases acidity, which may alter the metabolism of microorganisms in the rhizosphere (Wen et al. 2022). Some studies show that plants treated with different rhizobacteria increased exudate amino acid concentrations (Phillips et al. 2006). The concentrations observed in root exudates may indicate time-dependent modulation by rhizobacteria (Sasse et al. 2018). Soybean and rice plants, inoculated with *Pseudomonas* and *Rhizobium* rhizobacteria, respectively, showed the same exudation response (Kuzmicheva et al. 2017; Naher et al. 2008).

Sugars and amino acids exuded by roots can serve as carbon and nitrogen sources for rhizobacteria, facilitating colonization of both soil and roots (la Rosa et al. 2022; Wen et al. 2022). In our study, the observed reduction in total sugars and phenolic compounds in exudates from *Streptomyces*-treated plants could reflect several non-mutually exclusive mechanisms. First, PGPR are known to modulate plant root secretion patterns, including altered membrane permeability and expression of transporter proteins, leading to reduced exudation of specific compounds (Chaparro et al. 2013; Sasse et al. 2018). Second, rhizobacteria, including *Streptomyces*, can actively consume root-derived compounds, particularly sugars and certain phenolics, as substrates for growth and metabolism (Zhalnina et al. 2018; Dimkpa et al. 2009). Third, the production of auxins by *Streptomyces* strains—previously documented for CLV95 and CLV104 (Nozari et al. 2021; Franções et al. 2025)—may influence root exudation dynamics, as bacterial auxins can modulate plant carbon partitioning (Dimkpa et al. 2009; Macías-Rodríguez et al. 2018). Distinguishing between these possibilities would require complementary approaches, such as transcriptional analysis of root transporter genes or isotope tracing experiments to track carbon flux. Nevertheless, regardless of the primary mechanism, the net outcome, a shift in exudate composition toward increased amino acids and reduced sugars and phenolics, is consistent with the establishment of a beneficial rhizosphere interaction, as previously reported for PGPR-colonized plants (Sasse et al. 2018; Van der Meij et al. 2017). The increased exudation of total amino acids observed in *Streptomyces*-treated plants, particularly in CLV104, may reflect active modulation of plant nitrogen metabolism and secretion pathways. Amino acids released into the rhizosphere serve as both chemoattractants and nitrogen sources for beneficial bacteria, facilitating root colonization and sustained mutualistic interactions (Sasse et al. 2018; Wen et al. 2022; la Rosa et al. 2022). Similar increases in amino acid exudation have been reported in soybean and rice plants inoculated with PGPR, suggesting a conserved mechanism by which plants selectively recruit and support beneficial rhizobacteria (Phillips et al. 2006; Kuzmicheva et al. 2017). Carbohydrates released into the rhizosphere as exudates can be captured and used by microorganisms. The exudation of sugars in the rhizosphere favors plant-microorganism interaction and facilitates colonization by beneficial rhizobacteria (Hardoim et al. 2008). Tomato plants inoculated with *Trichoderma atroviride*, a beneficial soil fungus, show a reduction in the concentration of sugars in root exudates due to root colonization (Macías-Rodríguez et al. 2018). It is important to note that the exudate collection system used in this study represents a simplified environment that does not fully capture the complexity of soil-based rhizosphere conditions, including microbial interactions, nutrient gradients, and physical constraints. However, the use of short-term aqueous systems is a well-established approach for exudate studies, as it minimizes background interference and enables more accurate characterization of released compounds. In this context, our objective was not to replicate field conditions, but rather to compare relative differences in exudation profiles among treatments under standardized conditions. Additionally, although the transfer of plants from a solid substrate to water may induce transient physiological responses, all treatments were subjected to the same protocol, ensuring consistency across comparisons. Therefore, the results should be interpreted as treatment-dependent differences under controlled conditions rather than absolute representations of in situ rhizosphere processes.

Phenolic compounds root exudation represents an important strategy in the plant-soil interaction. Phenolic and flavonoid compounds are groups of specialized and ubiquitous metabolites in plants, presenting several functions in the plant-environment interaction, such as allelopathic action, chelators, and mediation of symbiosis with nitrogen-fixing rhizobacteria (Abdel-Lateif et al. 2012). Phenolic compounds exudated by roots have different activities in the soil, such as allelochemical activity (coumarins, coumaric and hydroxybenzoic acids) (Barkosky and Einhellig 2003; Blum et al. 1993; Razavi 2011). Other phenolics can interact with abiotic stresses such as caffeic and gallic acids, which enhance the response to oxidative stress such as in drought and salinity situations (El-Nagar et al. 2020; Riaz et al. 2018). Likewise, vanillic and ferulic acids can both interfere with plant oxidative stress responses and help mobilize soil nutrients (Ghareib et al. 2010; Mathew and Abraham 2004). Caffeic acid, like catechins, acts by reducing the respiratory rate of microorganisms present in the soil (Zwetsloot et al. 2018). Likewise, an increase in the concentration of caffeic acid in tobacco plant exudates was the main defense strategy against *Ralstonia solanacearum* (Li et al. 2021). The diversity of phenolic compounds exuded by roots can act as signals and modulators in the rhizosphere, favoring colonization by beneficial rhizobacteria and limiting the development of pathogenic microorganisms (Makoi and Ndakidemi 2007). In our work, the presence of *Streptomyces* caused a reduction in the concentration of phenolic compounds in the 15-day-old plant exudates, except in the CLV16 treatment, compared to the control. This reduction observed in maize plants contrasts with the increased exudation of phenolic compounds observed in *Cicer arietinum* plants inoculated with beneficial microorganisms such as *Trichoderma, Pseudomonas* and *Mesorhizobium* (Lobato Ureche et al. 2021). Likewise, *Capsicum annum* plants inoculated with *Cellulosimicrobium, Ochrobactrum, Enterobacter* and *Pseudomonas* increased the exudation of phenolics related to plant defense (Singh et al. 2014). Although the exudation of phenolic compounds by the roots is often associated with a plant defense mechanism against microorganisms, these metabolites also play important roles in plant–microbe interactions, including signaling and modulation of beneficial associations. Thus, the responses observed in plants treated with specific *Streptomyces* isolates may reflect a metabolic adjustment rather than a purely defensive reaction. The reduced exudation of total phenolics and specific defense-related compounds such as caffeic acid and coumarin in *Streptomyces*-treated plants has two non-mutually exclusive implications. First, it suggests that plants downregulate phenolic-based defenses upon recognition of beneficial bacteria, reallocating resources toward growth and mutualist support rather than antagonism (Sasse et al. 2018; Van der Meij et al. 2017). Second, lower phenolic concentrations in the rhizosphere may reduce selective pressure against sensitive microbial taxa, potentially promoting a more diverse or cooperative microbial community (Zhalnina et al. 2018). This interpretation aligns with our observation that CLV104, the strain inducing the lowest phenolic exudation, also promoted the highest amino acid release, suggesting a shift from defense to nutrient-based microbial recruitment.

Flavonoid root exudation plays a role in plant-microorganism communication, enabling symbiotic associations that favor plants (Phillips et al. 2006). Total flavonoids concentration in root exudates of 5- and 15-day-old plants (Fig. 5c, d) was higher in control plants, with no difference in treatment CLV95 5 days with control compared to *Streptomyces* treated plants.

Flavonoids are a diverse group of compounds belonging to the polyphenol class. Some of them, such as apigenin and quercetin, are associated with plant-mycorrhizal signaling in promoting the colonization of beneficial fungi in the rhizosphere, while rutin and kaempferol are related to the inhibition of phytopathogens (Cesco et al. 2012). In general, the profile of compounds exuded by roots is determined by the developmental stage, where more developed plants present less exudation of sugars and higher levels of amino acids and phenolics, as observed in Arabidopsis plants from 17 to 38 days after germination (Chaparro et al. 2013).

Phenolic and flavonoid metabolites are usually exudated by roots as part of the nutrient acquisition strategy (Iannucci et al. 2021). Caffeic acid is related to responses to biotic stresses, due to the concentration of rhizobacteria present in young roots (Riaz et al. 2018). In the present study, its occurrence in maize plants treated with *Streptomyces* isolates may be related to the interaction between the plant and rhizobacteria, especially in young roots, where microbial colonization is more active. Vanillic and ferulic acids are known antioxidants (Ghareib et al. 2010; Mathew and Abraham 2004) and occur in higher concentrations in CLV179 and CLV16 treatments respectively. Previous studies with CLV16 and CLV179 indicate that these rhizobacteria act by inhibiting the growth of maize plants (root dry mass decreased with CLV16, while shoot dry mass decreased with CLV179) (data not shown). The lack of detection of coumarins and coumaric acid, as well as the similarity of concentration between the *Streptomyces* treatments and the control, suggest a lack of allelopathy (Blum et al. 1993; Razavi 2011). Similarly, hydroxybenzoic acid is also related to allelopathic effects (Barkosky and Einhellig 2003), and was observed only in the control treatments (1.05 ng/mg) and in the CLV179 treatment (3.22 ng/mg).

Flavonoids analysed in this work (apigenin, kaempferol and quercetin) were previously related to plant-bacteria interaction, either as a signaling or inhibitory form (Cesco et al. 2012). Root exudation of rutin has been observed in eucalyptus plants in interaction with the mycorrhizal fungus *Pisolithus*, promoting both growth and orientation of hyphae (Lagrange et al. 2001). Rutin was the only flavonoid detected in the exudates of 5- and 15-day old plants. The other flavonoids evaluated in this work were not detected: apigenin, kaempferol and quercetin.

Among the phenolic compounds detected, caffeic acid, coumarin, and vanillic acid have recognized roles in plant–microbe interactions. Caffeic acid, for instance, has been shown to act as a chemoattractant for rhizosphere bacteria and to modulate redox status in root apoplast (Li et al. 2021). Conversely, coumarins are known to influence microbial community structure by inhibiting certain soilborne pathogens while sparing beneficial taxa (Razavi 2011; Stringlis et al. 2018). In the present study, the near-absence of coumarin and cinnamic acid in CLV104-treated plants, together with the marked reduction in caffeic acid exudation, suggests a strain-specific downregulation of phenylpropanoid-derived defense signals. Vanillic acid, which was highly exuded in CLV179-treated plants, has been implicated in both antioxidant activity and nutrient mobilization (Ghareib et al. 2010). Thus, the distinct exudation patterns observed among *Streptomyces* strains likely reflect divergent strategies of rhizosphere modulation, ranging from suppression of defense-related phenolics (CLV104) to maintenance of antioxidant phenolic signatures (CLV179). An important caveat in our exudate analysis is that we cannot definitively distinguish between active plant-driven alteration of exudation and microbial consumption or degradation of compounds during the collection period. Both mechanisms could contribute to the observed reduction in sugars and phenolics and the increase in amino acids. Future studies should employ sterile post-collection controls (to assess microbial consumption), isotope labeling (e.g., ^13^C-glucose or ^15^N-amino acids) to track carbon and nitrogen flux, and transcriptional analysis of root transporter genes to disentangle these processes. Nevertheless, regardless of the primary mechanism, the net outcome—increased amino acids and reduced defense-related phenolics—is consistent with the establishment of a beneficial rhizosphere environment, as previously described for PGPR-colonized plants (Sasse et al. 2018; Van der Meij et al. 2017).

Collectively, our results reveal a coordinated physiological response in maize colonized by *Streptomyces*, characterized by two interconnected features: attenuation of antioxidant enzyme activities (APX, CAT, POX) and alteration of root exudate composition (increased amino acids, reduced sugars and defense-related phenolics). These responses may be consistent with a transition from constitutive defense toward a more permissive mutualistic interaction. The early increase in POX activity (5 days) suggests an initial recognition phase, followed by downregulation of oxidative metabolism during the progression of the plant–*Streptomyces* interaction. Concurrently, the exudate shift toward primary metabolites likely may reflect increased availability of primary metabolites in the rhizosphere (Sasse et al. 2018; Van der Meij et al. 2017).

We acknowledge methodological limitations of the present study. First, inoculum concentrations differed among strains (ranging from 1.9 × 10^5^ to 7.6 × 10⁷ CFU/mL), as each strain was applied at its maximum growth under standardized culture conditions. Future dose–response experiments or CFU normalization will be needed to disentangle strain-specific properties from inoculum load effects. The strong physiological responses observed with CLV104, despite this variation, could potentially reflect either strain-specific biological properties or inoculum density-dependent effects. To distinguish between these mechanisms, future studies should employ standardized inoculum concentrations across all strains, allowing direct comparison of strain-specific traits independent of cell density. Alternatively, dose–response experiments using uniform strains with varied inoculum concentrations would clarify density-dependent versus strain-specific effects on plant physiology and exudate composition. While the current data suggest strain-specific effects (e.g., differential effects of CLV95 versus CLV179), we cannot exclude the possibility that inoculum density contributed to the magnitude of observed responses, particularly for CLV104. Second, we did not perform direct verification of root colonization (e.g., CFU re-isolation, microscopy, or molecular markers). Consequently, we cannot definitively confirm that all strains colonized roots with equal efficiency, nor can we determine whether differences in physiological responses reflect differential colonization and persistence versus intrinsic biological differences among strains. The observed strain-specific effects (e.g., contrasting responses to CLV104 versus CLV95) could potentially be explained by differential root colonization rather than strain-specific metabolic activity. Future studies should incorporate CFU enumeration, culture-independent quantification (16S rRNA gene qPCR), and microscopy-based visualization of root colonization to distinguish these mechanisms. Notwithstanding this limitation, the consistent coordination between enzyme attenuation and exudate compositional changes across multiple strains suggests a systematic plant response to *Streptomyces* inoculation, though the mechanistic basis remains to be fully resolved. Indirect evidence, reproducible strain-specific physiological responses across independent replicates and previous studies demonstrating effective maize root colonization by these isolates (Nozari et al. 2021), supports successful plant–bacteria interaction. Nonetheless, future studies should incorporate direct colonization assessments. Third, we acknowledge a critical ambiguity inherent to our experimental design: during the 24 h exudate collection period, *Streptomyces* strains remained present in the rhizosphere compartment. Also, prolonged aqueous immersion of roots can alter several physiological processes: (i) membrane permeability may be increased due to osmotic stress, leading to enhanced passive metabolite leakage rather than active exudation; (ii) hypoxia-related stress from anaerobic conditions in the waterlogged rhizosphere may alter ROS production and antioxidant enzyme activity; (iii) nutrient depletion in distilled water may trigger stress-responsive metabolic shifts; and (iv) the absence of soil-resident microbiota and dissolved nutrients contrasts sharply with natural rhizosphere complexity. These factors may collectively distort both enzyme activities and exudate profiles relative to field conditions. Future studies should employ soil-representative systems such as rhizotrons with controlled soil moisture, shorter collection windows (e.g., 6 h versus 24 h), or in situ metabolite collection methods (e.g., root xylem or phloem sap analysis) to better approximate natural rhizosphere metabolite dynamics. Nevertheless, the consistent strain-specific responses observed across our experimental conditions suggest that the observed effects reflect biological interactions, even if the magnitude or composition of measured metabolites may not fully recapitulate field conditions. This suggests that measured exudate profiles are likely to reflect net metabolite accumulation resulting from combined plant exudation, bacterial metabolite consumption, and microbial degradation of plant-derived compounds, not plant-driven processes in isolation. Consequently, we have revised our terminology throughout the manuscript to reflect this limitation. To distinguish plant-driven from microbial-driven metabolite changes in future studies, we recommend employing sterile exudate control treatments (plant root exudates collected in the absence of bacteria under identical environmental conditions) alongside inoculated treatments. This design would reveal the specific contribution of plant-driven exudation versus bacterial-mediated transformations and consumption. The current study characterizes the correlation between *Streptomyces* inoculation and coordinated changes in antioxidant enzyme activity and root exudate composition. However, we acknowledge that correlation does not establish causation, and our data alone cannot substantiate mechanistic interpretations regarding molecular signaling, transporter activation, or metabolic pathway modulation. Specifically, the study lacks: (i) direct ROS quantification via fluorometric or chemiluminescent assays to confirm oxidative stress status; (ii) transcriptomic analysis of plant defense and metabolic genes; (iii) quantification of bacterial signaling molecules (e.g., acyl-homoserine lactones or other quorum-sensing signals); (iv) direct measurement of metabolite fluxes using stable isotope tracing; (v) molecular characterization of root colonization and bacterial metabolic activity; and (vi) sterile exudate control treatments to partition plant-driven from bacterial-mediated metabolite changes. Considering these limitations, we have restricted mechanistic language throughout the revision, replacing causal or strongly directional terms (“orchestrates,” “modulates,” “triggers,” “induces”) with more conservative descriptors (“associated with,” “coincides with,” “changes in,” “alterations of”). This more restrained terminology reflects the confirmatory scope of our data: demonstrating associations and coordinated changes, not establishing mechanisms. Future experimental approaches integrating transcriptomics, ROS quantification, metabolic flux analysis, and direct visualization of bacterial colonization would be necessary to address the mechanistic questions raised.

This integrated response is likely involved with bacterial signals, including auxin production, which can modulate both plant carbon partitioning and defense signaling (Dimkpa et al. 2009). The strain-specific outcomes observed, particularly the pronounced effects of CLV104 on both enzyme attenuation and exudate alteration, underscore the importance of isolate selection for biotechnological applications. By linking oxidative defense modulation with exudate chemistry, our findings provide a more holistic view of plant: *Streptomyces* interactions and highlight the ecological relevance of metabolic integration in rhizosphere signaling. Although differences in inoculum concentration may influence plant physiological and metabolic responses, the results presented here were interpreted considering the overall functional performance of each isolate, integrating both biomass production and metabolite synthesis.

While previous studies have demonstrated that *Streptomyces* strains can alter root exudation or modulate oxidative stress individually (Van der Meij et al. 2017; Mattei et al. 2026), the present study provides three novel insights. First, it reveals a coordinated attenuation of multiple antioxidant enzymes (APX, CAT, POX) alongside a simultaneous alteration of root exudates toward primary metabolites, suggesting systemic integration of defense and exudation responses. Second, it demonstrates that strain-specific effects, particularly those of isolate CLV104, can strongly suppress the exudation of defense-related specialized metabolites (e.g., caffeic acid, coumarin) while enhancing amino acid release, a pattern not previously reported for maize–*Streptomyces* interactions. Third, it links these metabolic shifts to the functional classification of isolates as PGPR (CLV104) versus non-PGPR (CLV179), offering a functional framework for strain selection in biotechnological applications.

## Conclusion

This study demonstrates that *Streptomyces* colonization in maize induces a coordinated physiological response comprising (i) attenuation of APX, CAT, and POX activities, and (ii) alterations of root exudates toward increased amino acids and reduced defense-related phenolics and flavonoids. These findings support a model in which beneficial rhizobacteria actively suppress constitutive plant defense metabolism while promoting a more permissive, nutrient-based rhizosphere environment. The strain CLV104 emerged as a particularly promising candidate for biotechnological use, given its pronounced effects on reducing phenolic exudation (including coumarin and caffeic acid) and increasing amino acid release, while maintaining low oxidative enzyme activity. From an application perspective, CLV104-like strains may be suitable for formulating bioinoculants aimed at improving nutrient exchange and reducing metabolic defense investment in crops. Future research should focus on identifying the specific bacterial signals (e.g., auxins, secondary metabolites) responsible for these strain-specific responses and validating their effects under field conditions.

## Data Availability

The 16S rDNA sequences of the isolates were deposited in NCBI database GenBank under the following accession numbers: CLV16 (ON723945.1) (https://www.ncbi.nlm.nih.gov/nuccore/ON723945.1), CLV95 (MN461005.1) (https://www.ncbi.nlm.nih.gov/nuccore/MN461005.1), CLV104 (ON723950.1) (https://www.ncbi.nlm.nih.gov/nuccore/ON723950.1), and CLV179 (MN461009.1) (https://www.ncbi.nlm.nih.gov/nuccore/MN461009.1). Additional data are available upon request.

## Declarations

### Plant reproducibility

This study used seeds of maize (*Zea mays* L.), cultivar BRS 1015, a commercially available cultivar developed and distributed by Embrapa. The plant material was obtained through legal commercial channels and was cultivated under controlled experimental conditions. All procedures complied with Brazilian regulations and institutional guidelines for the use of cultivated plant species in research. No collection of plant material from wild populations was carried out; therefore, no specific permissions or licenses for plant collection were required.
